# On the Possible Overestimation of Cognitive Decline: The Impact of Age-Related Hearing Loss on Cognitive-Test Performance

**DOI:** 10.3389/fnins.2020.00454

**Published:** 2020-06-09

**Authors:** Christian Füllgrabe

**Affiliations:** School of Sport, Exercise and Health Sciences, Loughborough University, Loughborough, United Kingdom

**Keywords:** age-related hearing loss, hearing-loss simulation, cognitive performance, short-term memory, working memory, young normal hearing

## Abstract

Individual differences and age-related normal and pathological changes in mental abilities require the use of cognitive screening and assessment tools. However, simultaneously occurring deficits in sensory processing, whose prevalence increases especially in old age, may negatively impact cognitive-test performance and thus result in an overestimation of cognitive decline. This hypothesis was tested using an impairment-simulation approach. Young normal-hearing university students performed three memory tasks, using auditorily presented speech stimuli that were either unprocessed or processed to mimic some of the perceptual consequences of age-related hearing loss (ARHL). Both short-term-memory and working-memory capacities were significantly lower in the simulated-hearing-loss condition, despite good intelligibility of the test stimuli. The findings are consistent with the notion that, in case of ARHL, the perceptual processing of auditory stimuli used in cognitive assessments requires additional (cognitive) resources that cannot be used toward the execution of the cognitive task itself. Researchers and clinicians would be well advised to consider sensory impairments as a confounding variable when administering cognitive tasks and interpreting their results.

## Introduction

Over the past decades, there has been increasing interest in the role of cognition in (the decline of) speech processing across the adult lifespan (see Figure 1 in Füllgrabe and Rosen, 2016b). Indeed, even after controlling for factors affecting hearing thresholds and suprathreshold auditory processes (e.g., Humes et al., 1994; Füllgrabe et al., 2015; Johannesen et al., 2016), speech-in-noise perception remains largely variable among listeners. Idiosyncratic variability and ontogenetic declines in cognitive functioning (e.g., Salthouse, 2004) are likely to explain at least some of the unaccounted variance. Consequently, many studies in hearing science nowadays use inclusion or exclusion criteria based on performance in cognitive screening tests, and/or assess cognitive abilities as covariates when trying to explain speech-processing abilities. In clinical audiology, it is being debated (e.g., Shen et al., 2016) whether cognitive screening should be part of the standard assessment for a more individualized rehabilitation (American Speech-Language-Hearing Association, 2018). Finally, it is important to remember that cognitive testing constitutes the very basis of the study of the lifespan trajectory of cognitive abilities in healthy and pathological aging.

**Figure 1:**
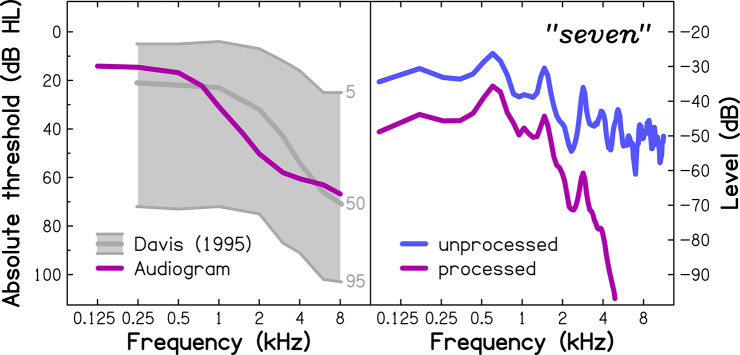
(Left panel) Extrapolated audiometric thresholds for an average 75-year-old listener (audiogram), used for the simulation of age-related hearing loss. For comparison, mean audiometric thresholds for 70- to 79-year-olds from a population-representative sample (Davis, 1995) are shown in the form of audiograms corresponding to the 5th, 50th, and 95th percentiles (light-gray lines). (Right panel) Average power spectra of the unprocessed (blue line; used with the NH group) and processed (purple line; used with the SHL group) spoken word “seven”.

Presumably for reasons of convenience and universal usability, most cognitive tests require the administrator to provide verbal instructions, and a substantial number use at least some auditorily presented stimuli (e.g., Mini Mental State Examination, Folstein et al., 1975; California Verbal Learning Test, Delis et al., 1987; auditory Stroop tasks, MacLeod, 1991). One such test is the Digit Span (DS) test, which, as part of well-established psychological test and screening batteries, such as the Wechsler Adult Intelligence Scales (Wechsler, 2008), the Wechsler Memory Scale (Wechsler, 2009), the Clinical Evaluation of Language Fundamentals (Semel et al., 2003), and the Montreal Cognitive Assessment (Nasreddine et al., 2005), is widely used by clinicians and researchers alike. The DS test comes in two versions: In the Forward DS (FDS) test, digit sequences spoken by the test administrator must be recalled in the order in which they were heard; performance on this test assesses short-term memory, i.e., the ability to temporarily store information. In the Backward DS (BDS) test, sequences of spoken digits must be recalled in reverse order; common wisdom assumes that performance on this test assesses working memory (WM), i.e., the ability to simultaneously store and process information (Baddeley, 1992), but empirical evidence indicates that the reordering of digits might require relatively little additional WM processing (Engle et al., 1999; Bopp and Verhaeghen, 2005). In contrast, complex span tests, such as the Sentence Span test developed by Daneman and Carpenter (1980), are designed to assess the key properties of the limited-capacity WM system, namely, memory storage and information processing. In the auditory version of this task, the so-called Listening Span (LS) test (e.g., Wingfield et al., 1988; Salthouse and Babcock, 1991; Lindenberger et al., 2001; Smith and Pichora-Fuller, 2015), lists of auditorily presented sentences must be processed (e.g., by judging the plausibility of the sentence), and information, provided during the presentation of the list (e.g., the sentence-final words), must be recalled at the end of the list.

A considerable number of hearing studies and most cognitive-aging studies include older participants, generally defined as over the age of 65 years (Erber, 2010). It is well known that sensory processing declines across adulthood, with one third of the over-65-year-olds being affected by disabling hearing loss (World Health Organization, 2019). However, hearing abilities of the study participants are either not assessed (as is the case in most cognitive-aging studies) or, if known (as is the case in most hearing studies), not necessarily considered when interpreting cognitive performance. Indeed, when the possibility of age-related hearing loss (ARHL) in the test sample is acknowledged by the authors, its impact on cognitive-test performance is either minimized (on the basis that “the experimenter raised their voice,” “the participants judged the used volume as adequate,” “the participants were asked to choose their preferred volume,” or “the participants wore their hearing aids”) or simply dismissed as warranting further investigation in future studies. Such methodological laxness is astonishing given the converging evidence that hearing impairment in older participants is associated with poorer cognitive performance (e.g., Rabbitt, 1991; McCoy et al., 2005; for a review, see Wingfield et al., 2005).

To further demonstrate the assessment-related impact of hearing loss on cognitive performance, the present study simulated audiometric and suprathreshold auditory processing deficits associated with ARHL in young normal-hearing participants and quantified their combined effect on memory span in several auditory-based memory tasks.

## Methods

### Participants

Fifty-six young (aged 18–22 years) native-English-speaking volunteers were recruited from the undergraduate student population of Loughborough University (United Kingdom), and received course credit for participating in the study. All participants completed the internet version of the hearing-screening test offered by the British hearing charity “Action on Hearing Loss” (https://www.actiononhearingloss.org.uk/hearing-health/check-your-hearing/) to establish that none had any hearing impairment. The test consists of listening to diotically presented triple digits (e.g., 6–2–5) in a fixed-level speech-shaped background noise (Smits et al., 2004) and varying the speech level to adaptively track the level for 50% correct identification. The test shows a high specificity (0.93) and test performance correlates strongly (*r* ~ 0.8) with the pure-tone average (PTA) for octave frequencies between 0.5 and 4 kHz (Smits et al., 2004). It is assumed that those who pass the test have a PTA of less than 23 dB HL, reflecting “good” hearing (Smits et al., 2006).

Participants were randomly assigned to one of two experimental groups: (i) 30 participants (77% females) with a mean age of 19.1 years [standard deviation (*SD*) = 1.1] formed the “normal-hearing” (NH) group that listened to unprocessed stimuli, and (ii) 26 participants (86% females) with a mean age of 19.3 years (*SD* = 1.3) formed the “simulated hearing loss” (SHL) group that listened to degraded speech. The small age difference between groups was not significant (Mann-Whitney *U*-test, *p* = 0.394, 2-tailed).

### Stimuli and Procedure

For the NH group, the stimuli were presented at 70 dB Sound Pressure Level. For the SHL group, the same sound-level settings were used, but the audio signals were processed using an algorithm developed by Nejime and Moore (1997) to simulate the following perceptual consequences of ARHL: (i) elevated hearing thresholds (by attenuating the frequency components in several frequency bands according to the threshold values given as an input); (ii) reduced frequency selectivity (by spectrally smearing the speech signal; Baer and Moore, 1994); and (iii) loudness recruitment (by expanding the range of the signal's envelope; Moore and Glasberg, 1993). The algorithm was implemented in a custom-written MATLAB program and received as its input the audiometric thresholds of an average 75-year-old listener (see left panel of Figure 1), as extrapolated by Fontan et al. (2017) from epidemiological audiometric data (Cruickshanks et al., 1998). The reason for choosing this particular age was that it falls centrally within the age range explored in many previous studies assessing older persons. The effect of the simulation on the power spectrum of the word “seven” is illustrated in the right panel of Figure 1.

The digit sequences for the two DS tests were taken from the Wechsler Adult Intelligence Scale - Third Edition (WAIS-III UK; Wechsler, 1997). In the FDS test, digit sequences of increasing length (from two to nine digits) were presented auditorily for immediate verbal recall. There were two trials for each sequence length. The final FDS score corresponded to the sum of recalled digits for all entirely correctly reported sequences; the maximum possible total score was 88. In the BDS test, digit sequences of increasing length (containing two to eight digits) had to be recalled in reverse order. The final BDS score was computed in the same way as the FDS score; the maximum possible total score was 70. An initial practice trial was given for each test.

For the LS test, short, grammatically correct sentences (e.g., “The ball bounced away”), taken from Rönnberg et al. (1989), were presented auditorily. Half of the sentences were sensible, whereas the others were absurd (e.g., “The pear drove the bus”). Sentences were arranged in sets of three to six sentences, with three trials per set length. The task was to listen to each sentence and then to indicate by a verbal “yes/no” response if the sentence made sense or not. At the end of each set, the participant was instructed to recall either the first or the last word of each sentence. The position (first or last) of the word to be remembered varied pseudo-randomly (with first-word recalls in half of the sets) but was identical for all participants. Prior to testing, practice was given in the form of one three-sentence set. The number of correctly recalled words in any order out of the total number of words to be recalled (i.e., 54) was taken as an estimate of WM capacity.

The timings used in the memory tasks were based on the rate of stimulus presentation recommended by the WAIS-III UK (Wechsler, 1997) for the two DS tests, or used by Rönnberg et al. (1989). The order of the tests was counterbalanced in the NH group and nearly counterbalanced in the SHL group. General test instructions were provided verbally by the experimenter at the start of each test. The test stimuli were recorded from an adult male native-British speaker with a standard British accent prior to the study, using a 44.1-kHz sampling rate with 32-bit quantization, and played diotically to the participant using the open-source audio software Audacity. All testing and the hearing screening took place in a quiet experimental room of the Sleep Laboratory of the School of Sport, Exercise and Health Sciences at Loughborough University, and used an HP (Palo Alto, CA) 250 G4 laptop, an external RME (Haimhausen, Germany) Babyface soundcard, and Sennheiser (Wedemark, Germany) HD580 headphones.

To investigate whether the simulation of hearing loss affected the intelligibility of the speech tokens, and thereby directly affected performance on the memory tasks, the SHL participants were asked, once the memory tests were completed, to listen to all digits and sentences once again and to repeat back what they had heard (in the case of sentences, only the first or last word of each sentence, which had to be recalled during the LS test, was scored).

## Results

The results for the three cognitive tests are given in Table 1. On average, raw scores for the SHL group were lower than those for the NH group, by 8, 10, and 10 percentage points for the FDS, BDS, and LS tests, respectively.

**Table 1:** Group-mean raw scores on the three cognitive tests for the normal-hearing (NH) and simulated-hearing-loss (SHL) groups, and statistical results from independent-samples *t*-tests (degrees of freedom, *df* ; *t*-value, *t*; *p*-value, *p*) for *z* score–transformed performance on each of the three memory tests.

**Cognitive measures**	**Listener group**		**Statistical results**		
	**NH**	**SHL**	***df***	***t***	***p***
Forward digit span (out of 88)	43.3	36.5	54	2.143	0.019
Backward digit span (out of 70)	32.3	25.6	54	2.652	0.005
Listening span (out of 54)	28.6	23.4	54	4.104	<0.001

*The maximum score is given in parentheses next to the name of the test*.

**Figure 2:**
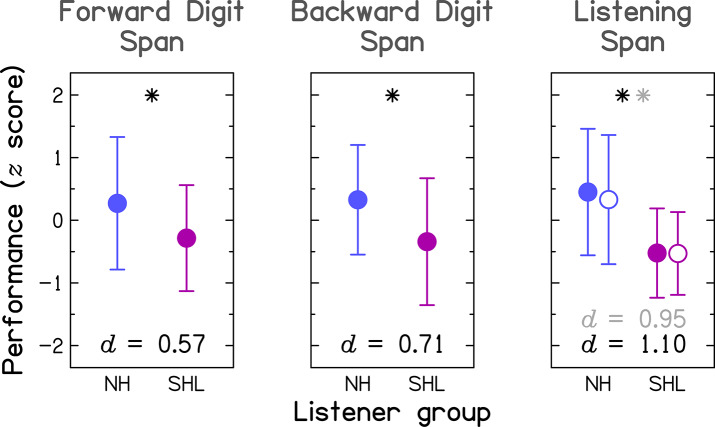
Group-mean performance (in *z* scores) between participants with normal hearing (NH; blue symbols) and participants with simulated hearing loss (SHL; purple symbols) for the three cognitive tasks. Error bars represent ±1 *SD*. The effect size is given by Cohen's *d* at the bottom of each panel. The asterisk indicates a significant group difference at *p* < 0.05. For the Listening-Span test, Cohen's *d* in light gray and the open symbols pertain to results obtained after excluding six SHL participants who did not show perfect intelligibility for the target speech.

To allow for the comparison across tests, the data were transformed into *z* scores, using the mean and the SD of the entire group (i.e., NH and SHL groups combined), prior to statistical analyses, and are shown in Figure 2. The effect size, expressed as Cohen's *d*^1^, was medium (0.5 ≤ *d* <0.8) for the two DS tests and large (*d* ≥ 0.8) for the LS test (see bottom of each panel in Figure 2). For all tests, performance differed significantly between the two listener groups, as indicated by independent-samples *t*-tests (all *p* ≤ 0.019, 1-tailed; for full results, see the three rightmost columns in Table 1).

The two listener groups also differed significantly in the number of errors on the semantic-judgment task of the LS test (Mann–Whitney *U*-test, *p* < 0.001, 2-tailed; data not shown), with the SHL group producing, on average, five times more errors (mean = 7.4) than the NH group (mean = 1.5).

All SHL participants correctly recognized the nine digits, but six of them (23%) failed to recognize two to four target words, representing 4 to 7% of the total number of words to be remembered in the LS test. Hence, all results of the LS test were reanalyzed, excluding those participants (see information in gray in the right panel of Figure 2). Although the value of the effect size was smaller compared to that observed when all SHL participants were considered, the effect size remained large (Cohen's *d* = 0.95). The group difference was still highly significant [*t*(48) = 3.30, *p* = 0.001, 1-tailed].

## Discussion and Conclusions

The observed results are consistent with the “effortfulness hypothesis” originally proposed by Rabbitt (1968, 1991)^2^ that suggests that, in suboptimal listening conditions (e.g., due to the presence of background noise or hearing impairment), the early stages of speech processing require additional efforts, thereby limiting the remaining cognitive resources available for the encoding in memory what was heard. Indeed, the present study found that the simulation of ARHL had an acute deleterious effect on the cognitive-test performance of young normal-hearing undergraduate university students, presumably free of cognitive impairment. For the here simulated level of ARHL, the reduction in forward and backward digit spans was not due to impaired intelligibility, as all participants in the SHL group were able to identify the processed test tokens. This observation could be interpreted as evidence that suprathreshold auditory processing deficits associated with ARHL alone cause a decline in memory performance. In the LS task, some participants of the SHL group were unable to recognize all to-be-remembered target words. Audibility or intelligibility of the other words of the carrier sentence could also have been suboptimal (but was not assessed in the present study). The higher number of errors in the simple semantic-judgment task found in the SHL group could be explained by such partial intelligibility of the sentences and/or insufficient cognitive resources available for the judgment task due to words being unintelligible or intelligible but degraded. Taken together, the results suggest that, when using auditory material to cognitively assess older persons who (un)beknown to the experimenter experience audiometric and/or suprathreshold auditory processing deficits, performance is likely to be underestimated.

The simulation used in the present study mimicked only some of the perceptual consequences associated with a moderate ARHL, namely, elevation of audiometric thresholds, loss of frequency selectivity, and loudness recruitment. There is currently no consensus on how to simulate changes in temporal processing abilities (Brian C. J. Moore, personal communication). However, there is converging evidence that sensitivity to temporal fine structure worsens with age and hearing loss (for a meta-analysis, see Füllgrabe and Moore, 2018) and that it plays a role in speech identification in quiet (Lorenzi et al., 2006) and in noise (Füllgrabe et al., 2015), possibly mediated via cognitive abilities such as selective auditory attention (Ruggles et al., 2012) and WM capacity (Füllgrabe and Rosen, 2016a). In addition, audiometric sensitivity further declines with age in the old-old and oldest-old, possibly at an accelerated rate (Göthberg et al., 2019). Both aforementioned caveats suggest that the present study underestimates the actual detrimental effect of auditory deficits on cognitive-test performance, especially in the oldest members of the population.

Qualitatively, the trends observed in the present study are in line with previous results, showing that signal attenuation (Lindenberger et al., 2001; Jorgensen et al., 2016), and background noise or other types of signal distortion (e.g., Rabbitt, 1968; Heinrich et al., 2008; Heinrich and Schneider, 2011) reduce the size of the memory span for auditorily presented speech tokens. Also, compared to age-matched normal-hearing controls, hearing-impaired persons perform lower on a variety of other auditory-based cognitive tasks (Rabbitt, 1991; van Boxtel et al., 2000; Dupuis et al., 2015), but it is unclear if this is due to auditory impairment affecting acutely the perceptual processing of test stimuli during cognitive assessment, or altering long-lastingly cognitive processing *per se*. Using an ARHL-simulation approach and young normal-hearing participants, the present study demonstrated that the former hypothesis is a possible, at least partial explanation for the apparently age-related cognitive decline that is observed when performance is assessed using auditory stimuli.

The reported findings have implications for the interpretation of previous and the design of future studies, assessing cognitive performance in unscreened older participants:

First, published reports of a worsening of performance on FDS and BDS tasks with age (e.g., Grégoire and Van der Linden, 1997; Myerson et al., 2003; Bopp and Verhaeghen, 2005) might at least partially reflect the consequences of ARHL on memory capacity, and less an age-related decline in the “true” ability to retain and process information. Consequently, models of cognitive aging, based on these data, probably overestimate the loss in memory capacity across the adult lifespan.

Second, the finding of a correlation between performance on auditory-based cognitive tests and performance on speech-identification tests in older people should not be interpreted as clear evidence for a cognitive involvement in speech processing, as this association could be caused, at least partially, by the deleterious effect of ARHL on performance in both tasks.

Third, future research assessing cognitive abilities should take into consideration the possible impact of ARHL on cognitive-test performance. This could be done by (ia) controlling experimentally (by including only participants with normal hearing functions) or (ib) statistically (by measuring sensory performance and using this information as a covariant) the effect of sensory decline, (ii) compensating for sensory deficits (by presenting stimuli at clearly suprathreshold levels, for example, by increasing the presentation level or by providing hearing aids to the participants), and (iii) using a visual version of cognitive tests originally including auditory-based tasks (e.g., the Montreal Cognitive Assessment for the severely hearing impaired, Lin et al., 2017). However, these alternative approaches come with their own limitations: (ia) as age-related sensory decline is ubiquitous, older persons with young-like sensory processing are hard to find, and their results are not representative of the general older population; (ib) statistically controlling for sensory deficits does not account for their effects over time in more central processing stations (Willott, 1996) that might negatively affect cognitive performance; (ii) the rehabilitative effect of amplification of the test material on cognitive performance has yet to be demonstrated (Saunders et al., 2018; Shen et al., 2020), as intelligibility of the test stimuli is not necessarily an issue (as shown in the present study), and high presentation levels (causing the broadening of the auditory filters and spectral smearing; Glasberg and Moore, 2000) and the use of hearing aids (resulting in the distortion of the speech signal; Moore, 2008) might impair speech processing and thus affect cognitive performance; and (iii) delivering instructions and test stimuli in the visual domain could still result in compromised cognitive performance as visual acuity also declines with increasing age (Gittings and Fozard, 1986; Klein et al., 1991).

Finally, as regards the clinical use of auditory-based screening and assessment tools (e.g., when diagnosing neurological disorders such as dementia, using the Hopkins Verbal Learning Test; Brandt, 1991), clinicians should be made aware of the risk of misdiagnosis associated with hearing impairment (Gates et al., 1996). For example, clinical-test manuals (e.g., Wechsler, 2008) could caution against the high prevalence of age-related auditory (and also visual) deficits in older test participants and their negative impact on cognitive-test performance. Routine assessment of sensory processing abilities in patients and participants would confirm the presence and severity of sensory impairments, but it is presently unclear how such additional information could be used to obtain a pure estimate of cognitive functioning [see issues discussed under (ib), (ii), and (iii) in the previous paragraph].

In conclusion, for older persons with sensory impairments, the perceptual processing of degraded internal representations of the test stimuli might require additional cognitive resources (Pichora-Fuller et al., 2016) that are unavailable for the execution of the cognitive task itself and, thus, compromise performance because of the limited nature of cognitive resources (Kahneman, 1973). This bias in cognitive-test performance might not be an issue when the aim of the cognitive assessment is to predict a person's real-life difficulties, as sensory deficits also play a role in everyday functioning (e.g., Brennan et al., 2005; Heyl and Wahl, 2012). However, establishing the relative contributions of sensory and cognitive factors to cognitive-test performance is crucial for the accurate diagnosis of the etiology of these difficulties and, thus, for their effective rehabilitation.

## Data Availability Statement

The datasets generated for this study are available on request to the corresponding author.

## Ethics Statement

The study was reviewed and approved by Loughborough University Ethics Approvals (Human Participants) Sub-Committee. The participants provided written informed consent prior to participation in the study.

## Author Contributions

CF designed the study, analyzed and plotted the data, and wrote the manuscript.

## Conflict of Interest

The author declares that the research was conducted in the absence of any commercial or financial relationships that could be construed as a potential conflict of interest.

## Footnotes

^1^Given the unequal sample size of the two listener groups, Cohen's *d* was calculated using the square root of the pooled variance rather than the mean variance (Howell, 2002).

^2^More recently, this notion has been discussed under the name of the “information-degradation hypothesis of aging” (Schneider and Pichora-Fuller, 2000; Wingfield et al., 2005; Wayne and Johnsrude, 2015).
